# Automated Joint Space Width Assessment in Patients Treated for Juvenile Osteochondritis Dissecans of the Distal Femur: A Cross-Sectional Study and Systematic Review of the Literature

**DOI:** 10.3390/jcm15041384

**Published:** 2026-02-10

**Authors:** Matthias Pallamar, Kaveh Same, Jennyfer Angel Mitterer, Sebastian Simon, Jan Philipp Nolte, Sebastian Farr, Jochen Hofstaetter, Catharina Chiari

**Affiliations:** 1 Department of Paediatric Orthopaedics and Foot Surgery, Orthopaedic Hospital Speising, Speisinger Straße 109, 1130 Vienna, Austria; sebastian.farr@oss.at (S.F.); catharina.chiari@oss.at (C.C.); 2 Michael Ogon Laboratory for Orthopaedic Research, Speisinger Straße 109, 1130 Vienna, Austria; kaveh.same@oss.at (K.S.); jennyferangel.mitterer@oss.at (J.A.M.); sebastian.simon@oss.at (S.S.); jochen.hofstaetter@oss.at (J.H.); 3 Center for Anatomy and Cell Biology, Medical University Vienna, Währinger Straße 13, 1090 Vienna, Austria; 4 Department of Neurology, Medical University of Vienna, Währinger Gürtel 18-20, 1090 Vienna, Austria; jan.nolte@meduniwien.ac.at; 5 2nd Department, Orthopaedic Hospital Speising, Speisinger Straße 109, 1130 Vienna, Austria

**Keywords:** juvenile, osteochondrosis dissecans, JOCD, OCD, knee, joint space width, JSW, systematic review, artificial intelligence, computer-aided detection, knee osteoarthritis

## Abstract

**Background/Objectives:** Juvenile osteochondritis dissecans (JOCD) of the knee is commonly treated using conservative or joint-preserving surgical techniques. While clinical outcomes are generally favorable, the risk of early cartilage degeneration remains unclear. Joint space width (JSW) on weight-bearing radiographs serves as an indirect marker of cartilage health. Artificial intelligence (AI)-based JSW assessment may enable sensitive and reproducible detection of early degenerative changes. **Methods:** This cross-sectional feasibility study included 21 skeletally immature patients treated for JOCD of the distal femur between 2002 and 2017. Treatment modalities comprised conservative management, retrograde drilling, and fragment refixation. Fully automated JSW measurements were performed on standardized anteroposterior knee radiographs using a validated AI-based software IB Lab KOALA™, Version 2.4. JSW of the affected compartment was compared with the contralateral knee and between treatment groups. Clinical outcomes were assessed using the Lysholm Knee Scoring Scale and the International Knee Documentation Committee (IKDC) score. Additionally, a systematic review of the literature on post-treatment degenerative changes following OCD therapy was conducted according to PRISMA guidelines. Results: Compared with manually reviewing images, the software IB Lab KOALA™, Version 2.4 as easy to implement. AI-based analysis revealed no significant differences in JSW between the affected and contralateral knees, nor between treatment modalities. Average JSW exceeded 6 mm in all groups after a median follow-up of 64 (min. 27, max. 177) months. Clinical scores were high and comparable across treatments. A moderate positive correlation was observed between the JSW and Lysholm score, while increasing age and longer follow-up were associated with a reduced JSW. The systematic review identified ten relevant studies, reporting generally favorable long-term clinical outcomes with a low but present risk of osteoarthritis progression. **Conclusions:** Our AI-based analysis showed no differences in JSW between conservative and joint-preserving surgical treatments of JOCD in the follow-up. This technology can provide a valuable tool for standardized and sensitive radiographic monitoring in young patients.

## 1. Introduction

Juvenile Osteochondritis Dissecans (JOCD) is described as focal alterations of the subchondral bone in skeletally immature patients. Its etiology has been related to impaired subchondral blood flow [1] with a possible contribution of repetitive microtraumas [2] and genetic factors [3].

The distal femoral condyle of the knee is the most prevalent JOCD location [4] and typically presents with poorly localized activity-related knee pain, sometimes associated with joint swelling and mechanical symptoms in later stages. Rare occurrence of JOCD has also been reported in the patella and the femoral trochlea [5].

The incidence of JOCD is estimated to be 6.8 to 11.2/100,000, most frequently presenting in children and adolescents aged 12 to 16 years. Males are more often affected than females (male/female ratio of 2–4:1) [6].

Restriction of sports activities and partial weight bearing as a non-operative treatment method [7], as well as surgical preservation techniques for drilling [8,9,10] or refixing [11,12,13], aim to completely heal the affected bone area and the adjacent joint cartilage, especially in skeletally immature patients.

However, it is unclear whether these preservation techniques can prevent the development of cartilage degeneration in the knee, which initially manifests itself radiologically as joint space narrowing and clinically as pain and functional impairment. The success of JOCD therapy can therefore be measured indirectly by recording the joint space width (JSW) and using clinical scores.

Conventional radiographs are widely used alongside Magnetic Resonance Imaging (MRI) for detecting joint degeneration and monitoring disease progression. Common scores, such as the Kellgren–Lawrence (KL) or OARSI scores, can be applied for this purpose. However, these scores show high interrater variability and low sensitivity for early detection of osteoarthritis. Artificial intelligence (AI)-based measurement of JSW can detect small changes that may be difficult to identify manually, which is particularly valuable for early degeneration detection in young patients.

The aim of the study is to determine whether artificial intelligence (AI)-based measurement of JSW also provides usable data for young patients after OCD therapy and to clarify whether three different forms of OCD-treatment (conservative, retrograde drilling and fragment refixation) result in differences in the JSW. The secondary aim was to assess correlations between the relative JSW and the Lysholm, as well as the International Knee Documentation Committee (IKDC) score.

To comprehensively compare the clinical results of our study with the scientific literature, we also conducted a systematic review on the topic of JOCD therapy and clinical and radiological evidence of the development of degenerative changes in the knee.

## 2. Material and Methods

This cross-sectional study was initiated in accordance with the Helsinki criteria following approval by the local ethics committee (EC number blinded for the review process). All study participants provided their written consent prior to the follow-up examinations. Between 2002 and 2017, 142 patients treated for distal femoral JOCD were analyzed from our prospectively maintained retrospective inhouse data registry.

All patients older than 16 years at the time of therapy, with bilateral knee lesions, previous operations of the knee, open knee surgery, any type of JOCD reconstruction, incomplete patient history, or missing bilateral standing long-leg radiographs (LLRs) at the last follow-up were excluded. Twenty-one patients were included for the final analysis, with a minimum follow-up time of 24 months.

The JOCD lesions were evaluated based on knee MRI examinations at the time of diagnosis. The median JOCD volume in patients treated non-surgically was 0.968 cm^3^ (min. 0.462/max. 2.772), while in the group treated with drilling it was 1.944 cm^3^ (min. 1.092/max. 7.315), and in refixed JOCDs, the median volume was 4.750 cm^3^ (min. 2.912/max. 8.140). Interruptions of the subchondral bone plate were seen in 1/3 of cases in the conservative and drilling groups. In refixed JOCDs the subchondral bone plate was interrupted in every case.

Our non-surgical treatment protocol for radiologically stable JOCD lesions, as assessed using magnetic resonance imaging according to Dipaola [14], consisted of a 4-week partial weight-bearing phase and an additional period of at least 2 months of abstinence from sports and physical activity. In cases of persistent knee complaints or instable lesions, knee surgery was performed. All patients undergoing surgery underwent diagnostic knee arthroscopy to assess the extent and stability of the lesion.

Stable JOCDs were drilled retrogradely (2.0 mm K-wires or 2.0 mm drills) under fluoroscopic guidance. Unstable JOCD lesions were fixed anterogradely with resorbable pins (1.5 mm Smart Nail^TM^ Conmed Linvatec Ltd., Tampere, Finland, Condral Dart^TM^, Arthrex Inc., Naples, FL, USA) or screws (2.7 mm Bio-Compressions Screws, Arthrex Inc., Naples, FL, USA). In all cases, a 6-week phase of partial weight-bearing followed the surgery.

All patients completed objective questionnaires, including an adoption of the Lysholm score [15] and the adjusted International Knee Documentation Committee Form [16].

**Figure 1:**
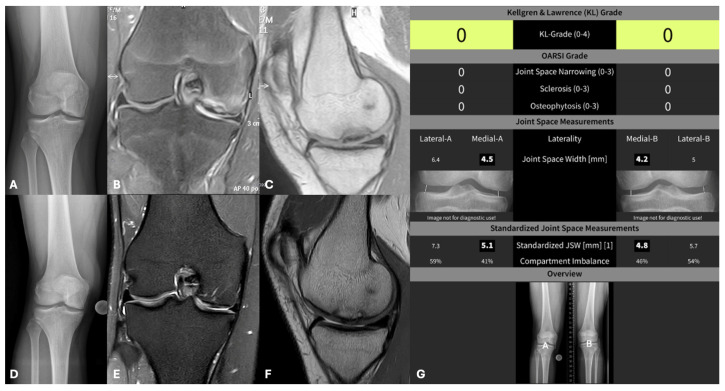
(**A**–**G**): Patient (16 years old) with OCD at the medial femoral condyle on the right knee before surgery, native X-ray (**A**); preoperative MRI scans (**B**,**C**); 6-year follow-up after OCD pinning with X-ray (**D**) and postoperative MRT scans (**E**,**F**); medial and lateral automatized joint space width evaluation of the right and contralateral left knee at defined measurement points (**G**).

Radiologic evaluation of anterior–posterior (ap) and lateral (weight-bearing position) knee radiographs was performed according to the hospital’s standardized protocol, with the patient in standing position and the patella facing to the front. The JOCD area was assessed for congruency, subchondral sclerosis, and partial dissection, as well as for the presence of osteophytic marginal attachments using ap and lateral radiographs. Bony healing was assessed using the available MRI examinations (Figure 1).

All ap knee radiographs used for JSW measurement were obtained in a bipedal standing position with the knee joints extended using digital X-ray (Digital Diagnost™ VM, Philips™, Eindhoven, The Netherlands), a focus-image receptor distance of 260 cm, and a scatter grid. The imaging technique ensured a centered patella position. The central beam was set at the level of the center of the knee joint.

The commercially available AI Software IB Lab KOALA™ version 2.4 (Knee Osteoarthritis Labeling Assistant, ImageBiopsy Lab™, Vienna, Austria) was used to perform automated measurements on each ap knee radiograph. The software was trained to measure knee joint space and to recognize osteoarthritic rim attachments to generate a Kellgren–Lawrence osteoarthritis grade. It was validated and trained on over 18,000 radiographs from the OAI (Osteoarthritis Initiative study; US six-site multi-center) [17], MOST (Multicenter Osteoarthritis Study, US two-site multi-center) [18], and other sites [19,20]. The software was run in a containerized environment using Docker on standard modern hardware with an Intel-i7 CPU and 32 GB of RAM, using the Ubuntu 22.04 operating system, with images stored on an external HDD. All images were manually checked by a trained orthopedic surgeon (MP).

We compared the JOCD lesions between three therapy groups, including non-surgical treatment, JOCD drilling, and JOCD fixation. The affected knee was compared with the contralateral side depending on the site of the JOCD lesion (medial or lateral).

Normally distributed variables are presented as means with standard deviation, whereas non-normally distributed variables are presented as medians with range. Two-group comparisons were performed using permutation-based Brunner–Munzel tests, and three-group comparisons were performed using the Kruskal–Wallis test. Categorical variables are shown as absolute and relative frequencies and were compared using Fisher’s exact test. To compare joint space width within participants, the JOCD knee was compared with the contralateral knee using a paired *t*-test. Pearson’s correlation was calculated to investigate associations between final OCD size and clinical outcome measures. Two-tailed *p*-values < 0.05 were considered statistically significant. All analyses were performed using R (version 4.1.0 or higher) and SPSS software (version 23.0; Chicago, IL, USA).

The systematic review section of this hybrid study was conducted in accordance with PROSPERO (international systematic review registry, study number CRD420251066512) guidelines and reported following the PRISMA (Preferred Reporting Items for Systematic reviews and Meta-Analyses) 2020 statement (Copyright © 2024–2025). Details on the PRISMA checklist can be found in Table S1 in the Supplemental Materials. The aim here was to collect data on radiological osteoarthritis development and clinical scores in connection with osteoarthritis development after joint-preserving therapy for OCD. A comprehensive literature search was performed across Medline, Embase, Web of Science, Scopus, and Google Scholar without time restrictions. References were managed using the Rayyan platform [21], where titles and abstracts were screened independently by two reviewers (KS and MP), followed by a full-text review of selected articles. Any disagreements during the screening process were resolved by discussion between the two. Eligibility was determined based on specific inclusion and exclusion criteria. Studies were included if they met the following criteria: (1) subjects had a confirmed diagnosis of osteochondritis dissecans (OCD) of the knee; (2) an intervention was performed; (3) the development of knee osteoarthritis was assessed via at least one designated outcome; and (4) the study design was a controlled trial, a prospective trial with historical controls, a prospective or retrospective cohort study, or a case–control study. Exclusion criteria comprised the following: (1) patients requiring knee intervention for reasons other than OCD; (2) the absence of any desired outcome data; (3) reconstruction procedures, including autograft and allograft implantations; and (4) non-analytic study types, such as case series, case reports, reviews, and conference abstracts. The collected outcomes included radiological measures, specifically the joint space width (mm) and Kellgren–Lawrence scores (Grades I–IV), as well as clinical functional and pain scores, such as the Lysholm Score, International Knee Documentation Committee (IKDC) Form, KOOS, and the Visual Analog Scale (VAS) for pain.

Data extraction was compiled into a master sheet capturing study characteristics, demographics, and complications. The risk of bias (quality) assessment was performed by one author (KS) and checked by another author (MP). The Cochrane Risk of Bias (RoB 2) tool was used for randomized controlled trials [22], while the ROBINS-I V2 tool was applied to non-randomized studies [23]. Additionally, the NIH Quality Assessment Tool for Before–After (Pre–Post) Studies with No Control Group was utilized for longitudinal studies [24]. Risk of bias visualization was obtained using the robvis online tool [25]. Data synthesis followed a narrative synthesis approach, augmented by clinical data from a cross-sectional study with longitudinal follow-up.

## 3. Results

Overall, 17 cases showed a lesion on the medial femoral condyle, and 4 cases involved a lesion on the lateral femoral condyle. At the time of follow-up, the mean patient age was 19.15 (±2.89) years, the mean BMI was 23.29 (±3.88), and the median follow-up duration was 64 months (minimum of 27 months, maximum of 177 months). All study patients included were distributed between the three treatment groups as follows: 4 patients underwent conservative treatment (non-surgical), 10 patients underwent retrograde OCD drilling, and 7 patients underwent OCD fixation. The results of the IKDC (mean 82 ± 15) and the LKSS (mean 88.29 ± 15.74) showed no significant differences between the groups. Baseline patients’ characteristics, clinical scores, and radiographic data in each treatment group are summarized in Table 1. In patients who underwent postoperative knee MRI, complete osteochondral healing of the former JOCD was observed in 8 out of 10 cases. Detailed data of the patient demographics are shown in Table 1.

The automated JSW measurement results were visually checked for plausibility in all radiographs, but no obviously incorrect measurement results were found in the outputs.

**Figure 2:**
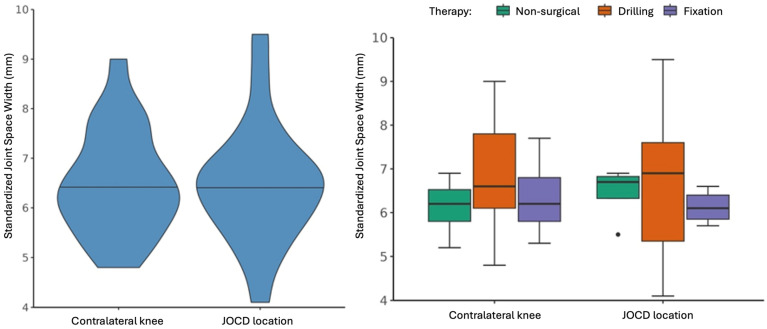
Standardized joint space width in mm between the OCD location and the contralateral knee (**left** panel). Standardized joint space width in mm by therapy type between the OCD location and the contralateral knee (**right** panel).

Table 2 and Figure 2 show the results of the automated JSW measurements between the therapy groups and between the affected JOCD knee and the non-affected contralateral knee. The measurements of the joint space revealed average values of over 6 mm in all groups directly above the former JOCD lesions, as well as in comparable joint compartments of the contralateral knee.

No significant difference in standardized JSW was observed between all affected JOCD knees and the non-affected contralateral knees (*t*(19) = −0.196, *p* = 0.846; mean difference = −0.04, 95% CI [−0.466, 0.386]).

The permuted Brunner–Munzel test showed no significant difference in standardized JSW between non-surgical and all surgical groups (*p* = 0.768), between the drilling and fixation groups (*p* = 0.312), or between the non-surgical and fixation groups (*p* = 0.339).

**Figure 3:**
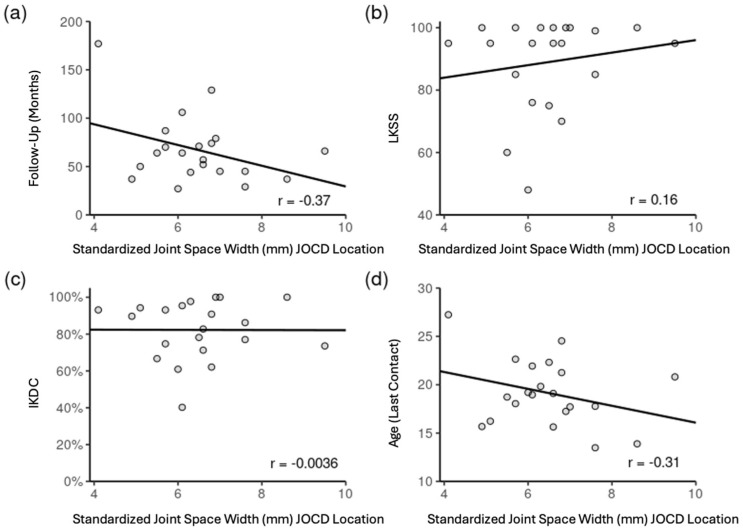
Correlations between standardized joint space width and the duration of follow-up (**a**), Lysholm Knee Scoring Scale (LKSS) (**b**), International Knee Documentation Committee (IKDC) (**c**), and patient age at time of follow-up (**d**).

We found a moderate indirect correlation between standardized JSW and patient age (r = −0.31) and length of follow-up (r = −0.37). In addition, there was a moderate direct correlation between the standardized JSW and the Lysholm Score (r = 0.16) (Figure 3).

**Figure 4:**
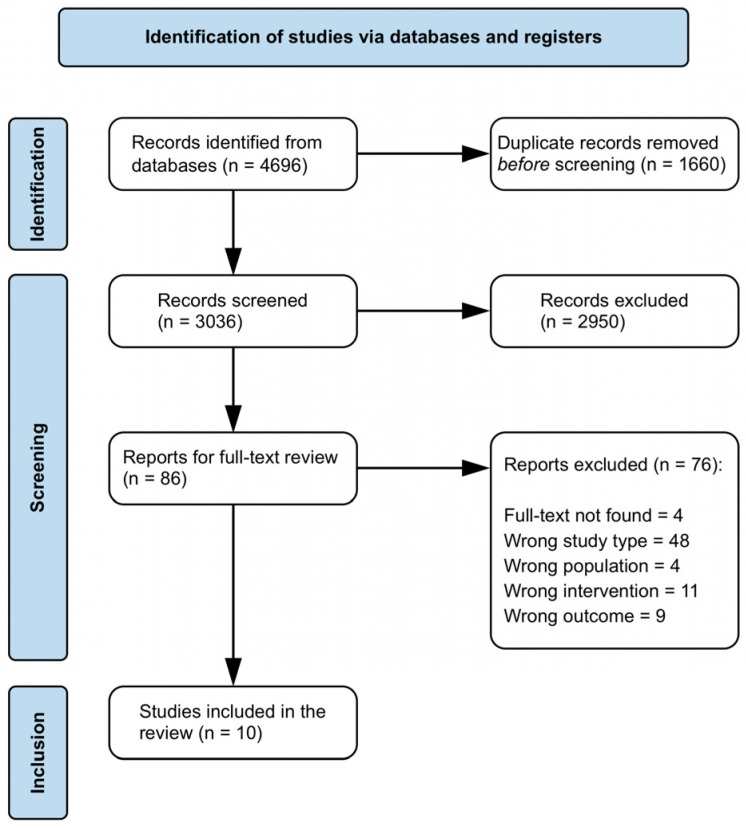
Systematic review flowchart diagram.

**Figure 5:**
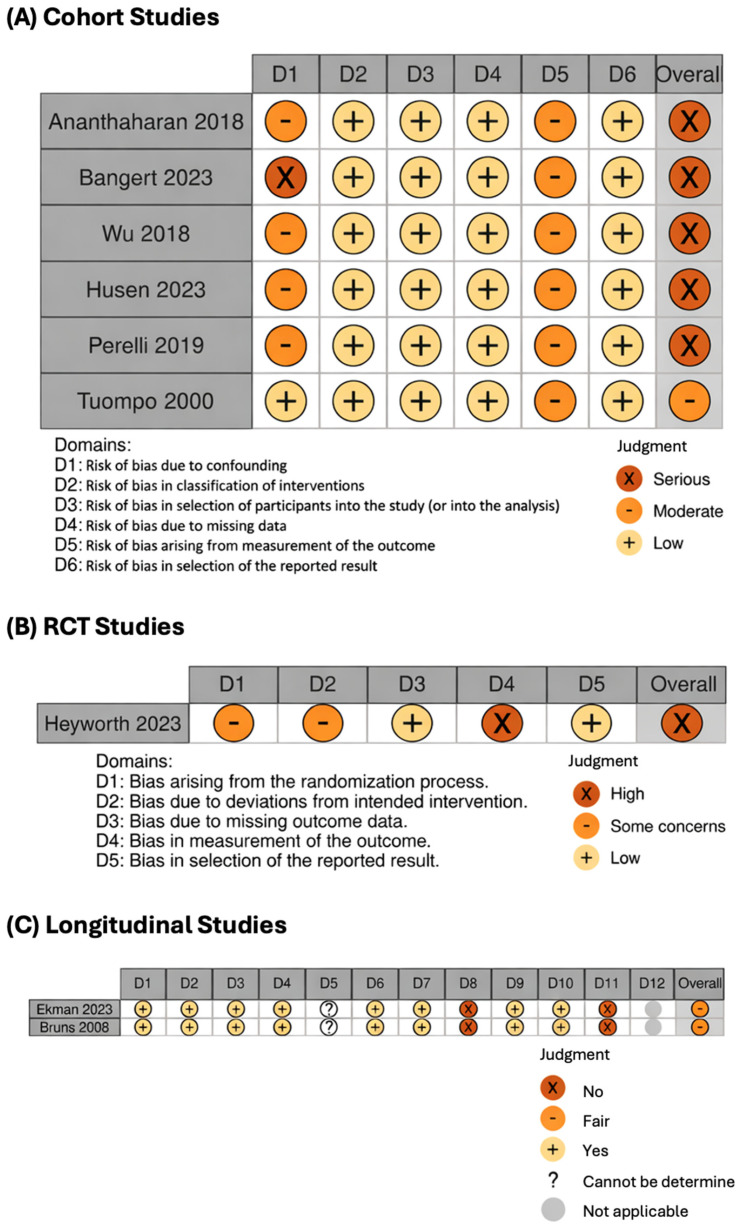
RoB plot. Items D1–12 for the longitudinal studies represent individual questions from the Quality Assessment Tool for Before–After (Pre–Post) Studies with No Control Group (https://www.nhlbi.nih.gov/health-topics/study-quality-assessment-tools, accessed on 9 September 2025), Ananthaharan 2018 is [26], Bangert 2023 is [27], Wu 2018 is [12], Husen 2023 is [28], Perelli 2019 is [30], Tuompo 2000 is [31], Heyworth 2023 is [32], Ekman 2023 is [33], Bruns 2008 is [34].

As part of our systematic review on radiological changes and function and pain scores after preserving OCD therapy, we were able to include 10 studies from an initial 4695 identifiable studies after the screening process (Figure 4). Of these 10 studies, 7 were cohort studies [12,26,27,28,29,30,31], 1 was an RCT [32], and 2 were longitudinal studies [33,34]. Details on the individual study quality assessment are provided in the RoB plot (Figure 5). A summary of the studies we were able to include in the review is presented in Table 3. Sample sizes vary widely, from 37 to over 100 patients, with most studies focusing on adolescents aged approximately 12 to 16 years.

## 4. Discussion

This study demonstrates that the use of automated JSW measuring software provides useful data for young adults after JOCD therapy. We found limited evidence that conservative and cartilage-preserving treatment methods for JOCD in the distal femur do not lead to differences in radiological JSW, either between treatments or compared with the contralateral knee. Furthermore, we found no significant differences in Lysholm and IKDC scores between conservative therapy, JOCD drilling, and JOCD fragment fixation in our patient collective. However, with increasing age, there is a symmetrical narrowing of the joint space and a decrease in the Lysholm score.

The Kellgren–Lawrence scoring system [35] evaluates radiological joint space narrowing only in later stages of osteoarthritis, although it must generally be assumed that the development of osteophytes and the reduction in cartilage thickness occur in parallel during the development of osteoarthritis. In this respect, the Osteoarthritis Research Society International (OARSI) Atlas classification should be given preference because it evaluates joint space narrowing and signs of osteophytes independently [36]. Early stages of osteoarthritis are connected to a reduction in cartilage width. Radiological joint space narrowing is one of the structural features in osteoarthritis grading according to the Kellgren–Lawrence [35] and OARSI Atlas grading systems [37]. Hence, measurements of the joint space width (JSW) in knee AP/PA radiographs are recommended to indirectly assess the cartilage width and progression to osteoarthritis [38]. In a study that assessed radiographic osteoarthritis progression, the mean estimated annual joint space narrowing rate was 0.13 +/− 0.15 mm/year [39]. Recently, automated computer measurements of JSW were presented [17].

There are advantages of AI-based compared with manual measurement of JSW on knee radiographs. AI-based methods provide highly consistent measurements, eliminating the inter-observer and intra-observer variability that commonly affects manual measurements. In addition, AI can also sensitively detect very slight changes in JSW, which could be particularly beneficial for young patients. The literature describes different fixed positions on measuring the JSW along the tibiofemoral joint [40,41]. The software used in this study calculates a relative standardized JSW, which allows for better differentiation between very mild consecutive OARSI grades and absolute fixed JSW [17]. This advantage is exploited in this study in young patients. In addition, this software has already been used to demonstrate a correlation between the severity of osteoarthritis and clinical scores [20].

To detect relevant changes in JSW in the knee over time of an individual patient, the smallest detectable change must be assessed. These cut-off values vary greatly from study to study, depending on the radiological positioning and the radiological procedure used [42]. In a systematic review of JSW cut-off values for detecting relevant osteoarthritis progression in the hip and knee, seven studies with values ranging from 0.22 to 0.78 mm were reported for the knee [43]. Since the differences in JSW between the JOCD knee joints and the contralateral knee in our clinical study are below these specified cut-off values, it can also be assumed that no cartilage degeneration will develop as a result of the different forms of JOCD therapy over our follow-up period.

Our clinical data show that conservative or surgical preservation treatments (through drilling and refixation) of the subchondral bone in the JOCD area do not cause JSW reduction of the overlying cartilage, which is consistent with long-term studies on these forms of therapy [29,33,34].

The results of our functional scores (Lysholm, IKDC) are also consistent with the literature in our review and confirm the absence of osteochondral changes in the treated joints. In our review, the Lysholm score showed consistently high results, with mean values ranging from approximately 76 to 92 points, and reported extremes spanning from about 70 to 100 points at follow-up. The IKDC subjective score, when reported, demonstrated mean values between 79 and 99, with minimum values around 62–70 and maximum values up to 100. The KOOS total or subscale scores (Symptoms, ADL, Sports, and Quality of Life) generally ranged from mean values of 55 to 94. Specifically, KOOS Symptoms and ADL domains showed higher means (approximately 85–94), while KOOS Sports and QoL were lower, with means commonly between 60 and 80. As in our work, the data show slightly poorer functional scores after JOCD refixation [12,26,27,30,31].

Our work also provides a cautious indication of a positive correlation between JSW and the Lysholm score. Correlations between JSW and KL osteoarthritis assessment using our measurement software and clinical scores, such as the Knee Injury and Osteoarthritis Outcome Score (KOOS), are described in the literature [20].

The performed systematic review included 10 other studies that also dealt with osteo-degeneration after JOCD therapy in the medium and long term. Functional outcome scores, such as Lysholm, IKDC, and KOOS are generally high across treatment modalities. Long-term follow-up studies reveal a low but notable risk of progression to osteoarthritis. Risk factors for poorer outcomes include greater lesion depth, lateral femoral condyle location, higher body mass index, older age, and operative treatment [12,33]. Due to the small number and heterogeneity of the included studies and their outcome measurements, it was not feasible to perform a meta-analysis.

There is not sufficient literature on the timing of joint degeneration in immature patients who have undergone non-operative treatment or surgical preservation of JOCD lesions of the knee. After a mean follow-up of 13.8 years, symptomatic osteoarthritis rates of about 8.0% and conversion rates to knee arthroplasty of less than 5.0% have been reported, regardless of whether initial management was surgical or non-operative [29]. In young adults treated non-operatively for knee JOCD, the cumulative incidence of moderate to severe knee osteoarthritis (Kellgren–Lawrence grade 3 or 4) was from 5.0% at 5 years to 30.0% at 35 years of follow-up [44]. In patients treated by fragment preservation, the cumulative incidence was 3.0% at 5 years, increasing to 51.0% at 30 years [45]. There is no data currently available regarding the early stages of cartilage degeneration in these patients.

In summary, the evidence for the treatment of JOCD of the knee is based predominantly on Level III studies. Retro- and transarticular drilling procedures are best supported by a Level I RCT. Conservative therapy shows good results in stable lesions, while advanced stages have a higher risk of failure. Good functional scores are generally reported after OCD fixation. However, there is no superior implant type, and fixations on the lateral femoral condyles have a higher risk of failure.

There are several limitations in this study. Our cross-sectional follow-up study design included different follow-up times and very different forms of treatment. However, in all of the patients included in this clinical study, the cartilage layer above the JOCD area was preserved. Surgical procedures that require cartilage reconstruction were excluded to keep the therapy groups comparable for JSW measurement.

Furthermore, we were unable to compare preoperative and postoperative X-ray images in this study because the preoperative X-ray images were not taken according to a standardized protocol. Only patients with unilateral JOCD were included in the study so that the healthy knee on the contralateral side could be used as a control for JSW measurements. However, the use of the opposite knee as a reference must be interpreted with caution, as we did not ask the study participants about their sporting habits (e.g., jumping leg or kicking leg), which could influence the results of the degree of cartilage wear.

Also, the software for automated measurement of the JSW was used in the study as an off-label application because it has primarily been validated in older populations. However, measurement of the JSW at the knee is based on the principle of geometric consistency at bony landmarks (femoral condyle and tibial plateau). At the time of JSW measurement, the patients’ growth plates were already closed and showed no anatomical changes in shape compared with older individuals. In addition, it can be assumed that in the absence of large osteophytes, the software can recognize the anatomical landmarks more precisely.

Lastly, given the exploratory design and limited sample size, particularly within certain subgroups, non-significant findings should be interpreted with caution, as small-to-moderate effects may not have been detected. However, the permutation-based and non-parametric methods used are well suited to small samples and distributional deviations. Future studies should replicate and validate the results from this study in larger, more homogeneous cohorts.

To our knowledge, this is the first study to assess the JSW after treatment of JOCD lesions in the knee joint using AI-based technology in young adults. Automated JSW measurement is technically feasible in this age group and provides useable data. However, validation studies of this technique in young age groups are still lacking.

## 5. Conclusions

Automated software procedures for monitoring JSW can be usefully employed in young adults following JOCD therapy. In this study, we found no differences in JSW following non-surgical treatment, drilling, or fixation of OCD.

In the conducted systematic review of signs of osteo-degeneration following OCD, treatment in adolescents and young adults, long-term follow-up studies reveal a low but notable risk of progression to osteoarthritis. Nevertheless, evidence for the development of osteoarthritis after OCD therapy is very limited.

## Figures and Tables

**Table 1:** Details of patients and therapy groups at the time of follow-up.

	Total	Non-Surgical	Drilling	Fixation	*p* Value
Patients (males/females)	21 (12/9)	4 (3/1)	10 (4/6)	7 (5/2)	-
Mean age (years) ± SD	19.15 ± 2.89	18.21 ± 2.39	18.63 ± 4.46	20.44 ± 1.83	0.173 ^¥^
Mean BMI ^1^ (kg/m^2^) ± SD	23.29 ± 3.88	23.02 ± 3.12	23.07 ± 4.28	23.74 ± 4.03	0.729 ^¥^
JOCD location (medial/lateral condyle)	17/4	2/2	9/1	6/1	-
Median follow-up in months (min/max)	64 (27/177)	69.00 (57/79)	47.50 (37/177)	70 (44/106)	0.516 ^¥^
Mean IKDC ^2^ (%) ± SD	82 ± 15	85 ± 14	87 ± 13	74 ± 19	0.298 ^¥^
Mean Lysholm ^3^ (%) ± SD	88.29 ± 15.74	88.75 ± 19.31	93.40 ± 9.37	82.71 ± 18.54	0.559 ^¥^
Radiographic congruency (convex/flat)	18/3	3/1	9/1	6/1	-
Radiographic subchondral sclerosis (no/yes)	18/3	3/1	9/1	3/4	-
Radiographic partial dissection (no/yes)	20/1	4/0	10/0	6/1	-
Radiographic osteophytic lipping (no/yes)	15/6	4/0	6/4	5/2	-
MRI (present/complete healing)	10/8	1/1	3/3	6/4	-

^1^ BMI indicates body mass index; ^2^ IKDC indicates International Knee Documentation Committee; ^3^ Lysholm indicates Lysholm Knee Scoring Scale (LKSS). ^¥^ indicates difference between the study groups.

**Table 2:** Results of relative joint space width at follow-up.

	Non-Surgical	Drilling	Fixation	*p* Value	
Standardized joint space width OCD location	6.45 ± 0.65	6.73 ± 1.70	6.13 ± 0.36	0.767 ^¥^ 0.321 ^†^ 0.339 ^‡^	0.846 ^1^
Standardized joint space width contralateral knee	6.12 ± 0.72	6.82 ± 1.35	6.34 ± 0.82	0.448 ^¥^ 0.442 ^†^ 0.797 ^‡^	

OCD indicates osteochondrosis dissecans; ^1^ difference between standardized joint space width OCD location and contralateral knee. ^¥^ difference between non-surgical and surgical treatment (drilling and fixation); ^†^ difference between drilling and fixation; ^‡^ difference between non-surgical and fixation.

**Table 3:** Summary systematic review.

First Author	Year	Study Type	Centers (n)	Cases (n = Knees)	Age at Therapy, Mean (±SD) or Median (Min–Max)	Group Characteristic (n)	Lesion Location (n) or (Rel %)	Follow-Up in Months, Median (Min–Max)	KL Score (n) or Tapper and Hoover (rel %)	Lysholm Score, Mean (IQR) or (±SD) or Median (Min–Max)	IKDC Form, Mean (±SD)	KOSS Pain, Mean (95% CI) or Mean (±SD)	KOOS Symptoms, Mean (95% CI) or Mean (±SD)	KOOS ADL, Mean (95% CI) or Mean (±SD)	KOOS Sports, Mean (95% CI) or Mean (±SD)	KOSS QoL, Mean (95% CI), Mean (±SD), or Median (Min–Max)	VAS, Mean (95% CI)	Notes	Level of Evidence	Justification for Evidence
Ananthaharan A. [26]	2018	Prospective and retrospective cohorts	1	87	12.7 (10–18)	ICRS I–II° (62)	Medial femur (63), 16 lateral femur (16), patella (6), trochlea (2)	48 (5–117)		84.0 (33)		82.1 (74.2–90.1)	58.1 (54.3–62.0)	88.9 (82.0–95.7)	69.0 (58.1–78.0)	66.3 (56.4–76.1)	2.0 (4)	Grades I–II lesions were successfully treated conservatively in 78% of cases. Grades III–IV lesions had a high failure rate		
						ICRS III–IV° (25)				80.0 (25)		78.9 (66.9–91.0)	55.9 (49.9–61.8)	89.3 (80.5–98.0)	62.4 (45.9–78.8)	67.3 (56.8–77.7)	2.0 (3)			
						Conservative treatment (49),				84.5 (27)		82.7 (73.6–91.8)	57.8 (53.5–62.1)	88.4 (79.5–97.3)	72.2 (60.1–84.4)	68.3 (57.5–79.1)	1.5 (3)		Level III	Large number of cases, but heterogeneous treatment arms
						drilling, fixation, debridement (38)				79.5 (37)		79.3 (69.7–89.0)	57.0 (52.0–62.0)	89.6 (83.7–95.6)	61.0 (47.6–74.4)	64.8 (54.5–75.0)	2.0 (4)			
Bangert Y. [27]	2023	Case–controls	1	37	14 (8–18)	Antegrade drilling (11),	Medial femur (24), lateral femur (7), patella (6)	168 (96–216)		91.7 (±9)	91.3 (±8.9)							Midterm results are sustainable and could improve further in the long term		
						fixation (10),														
						microfracturing (7),														
						autologous chondrocyte implantation (5),													Level III	Comparative study without randomization
						osteochondral autograft transfer system (1)														
Heyworth B. [32]	2023	Randomized controlled clinical trial	14	91	12.6 (±1,4) TAD 12.0 (±1.2) RAD	TAD transarticular drilling (51), RAD retroarticular drilling (40)		24		99 (91–100) TAD and 95 (86–100) RAD						94 (70–100) TAD and 81 (62–97) RAD		TAD resulted in shorter op/fluoro; better healing at 6 and 12 mo; earlier RTS; no PRO diff at 24 mo; secondary surgery rates of 4% vs. 10%	Level I	Multicenter RCT, direct therapy comparison (TAD vs. RAD)
Ekman E. [33]	2023	Retrospective cohort	1	103	21 (6–60)	Conservative treatment (24), drilling, fixation, debridement, microfracturing, autologous chondrocyte implantation, osteochondral autograft transfer system (79)		144 (84–240)	0° (39) I° (27) II° (22) III° (5) IV° (1)			KOOS total: 84 (77.0–88.0)						Risk factors for progression of osteoarthritis: lesion depth, higher BMI, higher age, operative therapy	Level III	Long-term follow-up, no control group
Wu I. [12]	2018	Cohort study	2	87	15.2 (±1.8) 20.8 (±6.0)	Internal OCD fixation in immature (27) and mature (60) patients	Medial or lateral femur	60 (24–166)			83.5 (±20.2)	89.7 (±14.1)	87.4 (±18.4)	93.9 (±12.7)	80.7 (±25.9)	78.9 (±23.1)		Lateral condylar location was an independent risk factor for failure (hazard ratio, 4.25; 95% CI, 1.47–19.85; P\0.01)	Level III	Multicenter, prospective/retrospective, no randomization
Bruns J. [34]	2008	Longitudinal study	1	42	15.9 (±2.8)	Immature (25) and mature (17) patients	Medial femur (33), lateral femur (9)	240 (120–300)	0° (46%) I° (26%) II° (14%) III° (11%) IV° (3%)	91.2 (±13.4)									Level III	Long-term data, no comparison group
Husen M. [28]	2023	Prospective and retrospective cohorts	2	80 (75)	15.2 (±1.9) and 21 (±6.1)	Bioabsorbable screws (20), metal screws (5),	Lateral femur (11), medial femur (13), trochlea (1),	136 (80–249)			86.6 (16.7)	88.7 +/− 18.1	89.3 +/− 12.6	89.3 +/− 21.6	79.8 +/− 26.3	76.7 +/− 26.3				
						Bioabsorbable screws (20), metal screws (36)	lateral femur (2), medial femur (41), trochlea (2)	137 (80–249)											Level III	Comparison of implant types, no randomization
Hevesi M. [29]	2020	Prospective and retrospective cohorts	2	95	12.8 (±1.9) and 12 (±2)	Fixation, drilling (25) allograft (1), palliative procedures (27),	Medial femur (69%), lateral femur (19.2%), patella (7.7%), trochlea (3.8%)		0° (91.4%) I–IV° (8.4%) 30-year-follow-up									I3 patients converted to total knee arthroplasty at a mean age of 51.5 (±2.6) years		
						Immobilization (7), brace (32), walking assistance (13),	Medial femur (97.6%), lateral femur (2.4%)		0° (100%) 30-year-follow-up										Level III	Long follow-up, but not controlled
Perelli S. [30]	2019	Prospective and retrospective cohorts	2	39	23.4 (±8.7) 20.1 (±5.1) 19.6 (±4.7)	Herbert screw	Lateral femur, (11) medial femur (28)	150		83.3 (±17.6)	79 (±16.2)							3 were performed using open approach, 6 did not heal		
						Cannulated screws		119		82.6 (±11.5)	77.1 (±13.2)							1 was performed using open approach, 2 did not heal	Level III	Small subgroups, no formal comparison
						Absorbable nail		94		82.4 (±11.1)	77.3 (±13.6)							5 were performed using open approach, 2 did not heal		
Tuompo P. [31]	2000	Prospective and retrospective cohorts	1	40	20.2 (±6.3) 23 (±8)	Bone peg	Medial femur (18)	103.6		76.2 (±18.9)								8 had normal knee, 5 had near normal knee, 4 had abnormal knee, 1 missing		
						Bioabsorbable rod	Medial femur (22)	82.1		84.2 (±13.5)								8 had normal knee, 9 had near normal knee, 5 had abnormal knee	Level IV	Small case series, older methodology

JOCD represents juvenile osteochondritis dissecans, ICRS represents International Cartilage Repair Society, KL represents Kellgren–Lawrence Score, IKDC represents International Knee Documentation Committee Form, KOSS represents Knee Injury and Osteoarthritis Outcome Score, VAS represents Visual Analog Scale for pain, TAD represents transarticular drilling, and RAD represents retroarticular drilling.

## Data Availability

The data that support the findings of this study are available from the corresponding author upon reasonable request.

## Supplementary Materials

The following supporting information can be downloaded at: https://www.mdpi.com/article/10.3390/jcm15041384/s1, Table S1: PRISMA Checklist 2020.
