# Supplementary material for: Automated Joint Space Width Assessment in Patients Treated for Juvenile Osteochondritis Dissecans of the Distal Femur: A Cross-Sectional Study and Systematic Review of the Literature
Source: J Clin Med. 2026 Feb 10;15(4):1384. doi: 10.3390/jcm15041384 (PMC12942276; doi:10.3390/jcm15041384)
Supplement: Supplementary file 1 [file jcm-15-01384-s001.zip › jcm-4092365-supplementary.pdf]

PRISMA 2020 Checklist

Section and Topic	Item #	Checklist item	Location where item is reported
**TITLE**			
Title	1	The title clearly identifies the report as including a systematic review.	Title page
**ABSTRACT**			
Abstract	2	The abstract reports background, objectives, methods, results, and conclusions in accordance with the PRISMA 2020 for Abstracts checklist.	Abstract section
**INTRODUCTION**			
Rationale	3	The rationale is described in the context of existing knowledge, highlighting uncertainty regarding long-term cartilage degeneration after JOCD treatment.	Introduction, paragraphs 1–5
Objectives	4	The objectives are explicitly stated: (1) to evaluate feasibility and results of AI-based JSW assessment after JOCD treatment, (2) to compare JSW between treatment modalities, and (3) to systematically review evidence on degenerative changes after JOCD therapy.	Methods – Systematic Review section
**METHODS**			
Eligibility criteria	5	Inclusion and exclusion criteria for the systematic review and the clinical study are clearly specified, including lesion type, treatment, outcomes, and study designs.	Systematic Review section
Information sources	6	Databases searched include Medline, Embase, Web of Science, Scopus, and Google Scholar. The date of last search is reported.	Methods – Systematic Review
Search strategy	7	A comprehensive multi-database search strategy without time restrictions is described.	Methods – Systematic Review
Selection process	8	Titles and abstracts were screened independently by two reviewers, followed by full-text review. Disagreements were resolved by discussion.	Methods – Systematic Review
Data collection process	9	Data extraction was performed using a predefined master sheet. Risk of bias assessment was conducted by one reviewer and verified by another.	Methods – Systematic Review

Section and Topic	Item #	Checklist item	Location where item is reported
Data items	10a	Radiological outcomes (JSW, Kellgren–Lawrence score) and clinical outcomes (Lysholm, IKDC, KOOS, VAS) are clearly defined.	Methods – Systematic Review
	10b	Additional variables include age, lesion location, treatment type, follow-up duration, and BMI.	Methods – Systematic Review
Study risk of bias assessment	11	Risk of bias was assessed using RoB 2 for RCTs, ROBINS-I for non-randomized studies, and the NIH tool for before–after studies.	Methods – Systematic Review
Effect measures	12	Effect measures included JSW in millimeters, KL grades, and standardized clinical score values.	Methods – Systematic Review
Synthesis methods	13a	Studies were grouped by treatment modality and outcome type.	Methods – Systematic Review
	13b	No statistical data transformation was required; reported outcomes were extracted as published.	Methods – Systematic Review
	13c	Results are presented in structured tables and figures, including PRISMA flow diagram and ROB plots.	Results – Figures 4–5, Table 3
	13d	A narrative synthesis approach was used due to heterogeneity of study designs and outcomes. No meta-analysis was performed.	Results – Systematic Review
	13e	Heterogeneity is discussed qualitatively based on lesion characteristics, treatment type, and follow-up duration.	Discussion
	13f	No sensitivity analyses were conducted due to the narrative synthesis approach.	Not applicable
Reporting bias assessment	14	Formal assessment of reporting bias was not performed due to limited number and heterogeneity of studies.	Not applicable
Certainty	15	Certainty of evidence was assessed qualitatively based on study design, risk of bias, and	Discussion

Section and Topic	Item #	Checklist item	Location where item is reported
assessment		consistency of findings.	
**RESULTS**			
Study selection	16a	The number of identified, screened, excluded, and included studies is reported using a PRISMA flow diagram.	Results – Figure 4
	16b	Reasons for exclusion are described at the full-text screening stage.	Results – Systematic Review
Study characteristics	17	Characteristics of all included studies are summarized in a comprehensive table.	Results – Figure 4
Risk of bias in studies	18	Risk of bias assessments are presented graphically.	Results – Figure 5
Results of individual studies	19	Key radiological and clinical outcomes are reported for each study.	Results – Table 3
Results of syntheses	20a	Included studies generally report favorable clinical outcomes with low but present risk of osteoarthritis progression.	Results – Systematic Review
	20b	No statistical synthesis or meta-analysis was conducted.	Not applicable
	20c	Potential sources of heterogeneity are described narratively.	Discussion
	20d	No sensitivity analyses were performed.	Not applicable
Reporting biases	21	Risk of reporting bias is discussed qualitatively.	Discussion
Certainty of evidence	22	Overall certainty is discussed as moderate to low due to predominance of Level III evidence.	Discussion
**DISCUSSION**			
Discussion	23a	Results are interpreted in the context of existing literature.	Discussion
	23b	Limitations of the included studies are discussed.	Discussion
	23c	Limitations related to heterogeneity and lack of meta-analysis are acknowledged.	Discussion
	23d	Implications for clinical practice and future research are outlined.	Discussion

Section and Topic	Item #	Checklist item	Location where item is reported
**OTHER INFORMATION**			
Registration and protocol	24a	The systematic review was registered in PROSPERO (PROSPERO 2025 CRD420251066512).	Methods – Systematic Review
	24b	A protocol was prepared and registered; amendments are reported.	Methods – Systematic Review
	24c	No major deviations from the protocol are reported.	Methods – Systematic Review
Support	25	Sources of support are declared.	Funding / Acknowledgments
Competing interests	26	Competing interests are declared.	Conflicts of Interest section
Availability of data, code and other materials	27	The data that support the findings of this study are available from the corresponding author upon reasonable request.	Data Availability Statement

Table S1: Supplementary PRISMA 2020 Checklist
"Automated joint space width assessment in patients treated for juvenile osteochondrosis dissecans of the distal femur: A feasibility study and systematic review on knee cartilage degeneration"
